# Enhancing IoT botnet detection with explainable ensemble learning

**DOI:** 10.3389/fdata.2026.1811967

**Published:** 2026-08-12

**Authors:** Linda Joseph, Sambath M., Vivekanandan M.

**Affiliations:** School of Computer Science and Engineering, Vellore Institute of Technology, Chennai, India

**Keywords:** botnet detection, cybersecurity, ensemble learning, Explainable AI, Internet of Things, intrusion detection, network security, SHAP

## Abstract

**Introduction:**

Internet of Things (IoT) botnet detection faces significant challenges due to the growing intricacy and decreased transparency of Machine Learning (ML) models.

**Methods:**

In this work, we provide an ensemble-based detection system that makes use of a voting classifier made up of a Boosted Decision Tree and a Bagged Random Forest. Using manual feature extraction, the model is trained and assessed using the N-BaIoT dataset. The popular Explainable AI (XAI) method, SHapley Additive exPlanations (SHAP), is used to analyze feature contributions across models while taking important factors like consistency, sensitivity, and monotonicity into account in order to improve the interpretability of the features. SHAP eases the black-box characteristic of sophisticated machine learning models by quantifying the influence of specific features, hence facilitating transparent model interpretation.

**Results:**

According to experimental data, the ensemble model is more sensitive than individual classifiers. Additionally, dynamic changes in SHAP values are shown by the weight adjustments made within the voting classifier, highlighting the impact of weight tuning on feature importance.

**Discussion:**

This study highlights the effectiveness of integrating SHAP-based XAI into ensembled models, enhancing the transparency, interpretability, and reliability of IoT botnet detection systems.

## 1 Introduction

The Internet of Things (IoT) has revolutionized a number of industries by allowing billions of related devices to exchange data across manufacturing, transportation, healthcare, and smart homes. One well-known instance is the Mirai botnet, which was initially identified in August 2016 and used IP cameras and DVRs with frail credentials to enslave more than 400,000 devices. IoT devices are still quite vulnerable to botnet assaults, nevertheless, because to their limited computing power, non-standard defenses, and inadequate security measures. The notorious Brian Krebs website attack, which peaked at 620 Gbps, was one of the several huge Distributed Denial of Service (DDoS) operations that Mirai unleashed (Kambourakis et al., 2017).

Botnets, which are networks of infected devices managed through Command and Control (C&C) channels, carry out advanced cyberattacks such as spam campaigns, malware distribution, DDoS flooding servers, and phishing to steal data. Notably, botnets like Rustock and Cutwail account for approximately 80% of global spam email traffic (Asadi et al., 2024). Their rapid spread in IoT environments, exploiting weak security, heightens risks. As per the survey, botnet detection faces challenges from evolving tactics like encryption, necessitating advanced strategies to secure IoT ecosystems (Asadi et al., 2024).

IoT botnet activity has significantly increased in recent years, with hackers consistently using old firmware, default settings, and lax authentication to infiltrate devices. In 2024 alone, IoT-driven DDoS traffic surged by 25%, fueled by profit driven hacking groups targeting smart homes, industrial IoT (IIoT) systems (USTelecom, 2024), and critical infrastructure. Malicious botnet activity exceeded one million infected devices between December 2023 and January 2024 (Infosecurity Magazine, 2024), with state-sponsored cyber actors contributing to the expansion of large-scale botnets. Additionally, key IoT devices with vulnerabilities in denial of service include smart TVs (36.7%), smart plugs (22.2%), digital video recorders (DVRs) (17. 7%), routers (13. 4%) and set-top boxes (6.9%) are among the major IoT devices that have denial of service vulnerabilities (Bitdefender, 2024). These increasing dangers emphasize how urgently sophisticated security frameworks that can defend IoT systems from changing cyberattacks are needed.

A variety of techniques, ranging from conventional approaches to advanced Deep Learning (DL) models, have been developed to detect and mitigate IoT botnet attacks. Anomaly-based detection methods identify deviations from normal network behavior but are often prone to high false positive rates. In contrast, signature-based approaches effectively detect known attack patterns but struggle to generalize to previously unseen botnet variants.

In recent years, Machine Learning (ML) and Deep Learning (DL) models have gained significant attention due to their ability to improve detection accuracy. Models such as Recurrent Neural Networks (RNNs) and Convolutional Neural Networks (CNNs) can capture complex temporal and spatial patterns in network traffic. More advanced approaches, including Generative Adversarial Networks (GANs), have been employed to generate synthetic attack data, further enhancing detection performance.

Despite their effectiveness, DL-based models have notable limitations. Their applicability in resource-constrained IoT environments is often restricted due to high computational requirements, potential overfitting, and their inherent black-box nature, which limits interpretability and makes it difficult to understand model decisions (Al-Shurbaji et al., 2025).

The increasing complexity of Deep Learning (DL) models has made them highly effective but opaque, often referred to as black-box models. This lack of interpretability has raised concerns, particularly in mission-critical applications that involve health- care, finance, and cybersecurity, where transparency is essential. To improve AI model transparency and interpretability, Explainable Artificial Intelligence (XAI) methodologies have been presented. Popular XAI methods include LIME and SHapley Additive Explanations. LIME creates locally faithful surrogate models that approximate black- box model predictions by perturbing data around a specific instance and training a simpler interpretable model. This clarifies the model's localized decisions. Game theory gives characteristics Shapley values to represent model prediction contribution and enable global interpretability in SHAP. SHAP is popular for interpreting complicated AI models because it provides more consistent and theoretically informed explanations. While DL models typically achieve higher accuracy, they often lack transparency. In contrast, ensemble techniques, that involve RF and Gradient Boosting, tend to generate more interpretable decision-making processes, making them better suited for security- critical applications. Ensemble models appear to offer better interpretability compared to intricate Deep Learning (DL) models (Hassija et al., 2024).

To enhance the precision and resilience of IoT botnet detection, we used an Ensemble Voting Classifier in this work that included Boosted Decision Trees and Bagged Random Forest. We performed a SHAP analysis to interpret the model's decision-making process and the features' contribution. This required assessing both the ensemble configuration and the individual models under various weight distributions. We were able to evaluate each feature's contribution to the model's predictions by using SHAP, which gave us important information about how important different traits are. Additionally, the feature set used in the ensemble model was aligned with the base paper's feature selection criteria, ensuring consistency and comparability with previous research.

## 2 Literature survey

The IIoT powers efficient production by linking smart devices through Information-Centric Networking (ICN), which shares data for clever operations but struggles with botnet attacks and unauthorized network entries. Their Checkpoint-Intrigued Adversary Mitigation Scheme (CIAMS) tackles botnets with smart learning, cutting false alarms by 10.35% and boosting service flow and efficiency by 11.68% and 12.55%, respectively (Kalakoti et al., 2022). Meanwhile, the “Variant Loop Detection and Mitigation Model (VLDMM)” uses recurrent learning to spot disruptions by reviewing past logs, keeping production smooth by adapting to control shifts (Coroama and Groza, 2022). With the rapid growth of IoT devices, advanced AI techniques have become essential for detecting botnets and malware more effectively. Researchers have focused on key areas like network traffic analysis. Swathi et al. (2024) proposed a framework, which combines traffic-flow metrics (e.g., session waiting time, bandwidth consumption) with botnet- specific features (e.g., packet count, flow duration). The model uses fuzzy clustering and a voting-based ensemble classifier to improve accuracy and effectively predict the severity of botnet attacks (Donald and Joseph, 2024).

Ensemble learning techniques, particularly gradient boosting, have demonstrated promising performance in addressing dataset imbalance and improving the precision of IoT malware detection (Donald and Joseph, 2023). Additionally, Sharma et al. (2024) emphasized the growing significance of XAI frameworks, which contribute to the transparency and reliability of DL models, that is important for fostering trust in automated detection systems (Swathi et al., 2024). The effectiveness of a number of machine learning classifiers, that include Naive Bayes, “K-Nearest Neighbors (KNN)”, “Support Vector Machines (SVM)”, and decision trees, for IoT botnet detection was assessed by Alshamkhany et al. (2020). The study made use of the UNSW and Bot-IoT databases, which included a variety of botnet attack patterns and a large amount of real-world IoT traffic. Decision tree-based classifiers have been widely recognized for their strong predictive performance and effectiveness in IoT botnet detection tasks.

Aljabri et al. (2024) introduced a hybrid classification approach by integrating XGBoost with other models to enhance ransomware detection, achieving 97.85% accuracy with only 47 selected features and a 2% false positive rate, underscoring the potential of memory dump analysis in ransomware identification (Sharma et al., 2024). Oppong et al. (2021) introduced an Improved Botnet Attack Detection framework by employing “Principal Component Analysis (PCA)” and an Ensemble Voting Algorithm, achieving 99.79% accuracy using Naive Bayes and KNN (Alshamkhany et al., 2020).

Alenezi and Ludwig (2021) used SHAP to understand ML models for cybersecurity threat detection using Explainable AI (XAI). XGBoost obtained the highest accuracy with 3,722 properly identified samples when Random Forest, XGBoost, and Keras Sequential classifiers were applied to datasets of malicious URLs and Android malware (Hassija et al., 2024). Wang et al. (2020) proposed an Explainable ML Framework for IDS that employs SHAP to deliver local and global explanations to improve transparency as well as interpretability (Aljabri et al., 2024).

Kalakoti et al. (2024) use the N-BaIoT, MedBIoT, BoT-IoT datasets to detect IoT botnets and attain F1-scores of above 99% using XGBoost. By using LIME as well as SHAP to produce interpretable explanations, their work ensures that security analysts can trust and comprehend model decisions. These explanations are thoroughly evaluated using criteria like faithfulness (0.907 for SHAP), monotonicity, complexity, and sensitivity (Gummadi et al., 2024). Tabassum et al. (2022) focus on a broad spectrum of IoT attacks, including DDoS and ransomware, using the ToN IoT dataset. They integrate LIME, SHAP, and ELI5 to deliver accessible and intuitive explanations, achieving over 96% accuracy with models like Random Forest and Decision Tree, making their approach valuable for diverse IoT environments (Alenezi and Ludwig, 2021). Wang et al. (2020) pioneer the application of SHAP for intrusion detection systems, applying it to the NSL-KDD dataset to achieve 80.6% accuracy. Their work lays a foundational framework for transparent intrusion detection, emphasizing feature-attack relationships (Oppong et al., 2021). Heydari and Nyarko (2024) concentrate on ransomware detection, analyzing a 62,485-sample dataset with SHAP and GANs to achieve an impressive 99.99% accuracy. Their emphasis on fair- ness through GAN integration ensures equitable model performance, addressing ethical concerns in security applications (Wang et al., 2020). Collectively, these efforts advance IoT security by combining high-performance detection with clear, trustworthy explanations, fostering confidence in automated systems. Gummadi et al. (2024) propose the XAI- IoT framework in their IEEE Access paper, enhancing anomaly detection in smart manufacturing (MEMS dataset) and IoT security (N-BaIoT dataset). Their frame work integrates seven XAI approaches—SHAP, LIME, LOCO, CEM, ALE, PFI, and Weight for global and local feature importance analysis, identifying critical features like Z-axis vibration (MEMS) and HH L1 magnitude (N-BaIoT). Its open-source release and evaluation across DNN and Random Forest models set a scalable benchmark for predictive maintenance and cybersecurity (Tabassum et al., 2022). Using ensemble learning (Random Forest, XGBoost, Extremely Randomized Trees, Histogram-based Gradient Boosting) and feature selection via Random Forest Feature Importance and SHAP XAI for transparency, Adriansyah et al. (2024) propose an Android malware detection framework that achieves 94.88% accuracy on the CICMalDroid2020 dataset. Their methodology prioritizes model interpretability and feature relevance (Heydari and Nyarko, 2024). The SPIP framework for IoT IDS is presented by Keshk et al. (2023). It combines XAI approaches (SHAP, PFI, ICE, and PDP) with “Long Short-Term Memory (LSTM)” to offer both local as well as global explanations, improving interpretability and trust. The NSL- KDD, UNSW-NB15, and Tonne IoT datasets were used for validation. It decreases training time and increases detection accuracy. In Android and IoT environments, both studies emphasize how important XAI is for promoting trust, facilitating effective cyber defense, and making complex ML/DL models transparent.

## 3 Proposed methodology

This section presents the proposed methodology in detail. The entire module of suggested scheme is presented in detail. an ensemble-based detection system that makes use of a voting classifier made up of a Boosted Decision Tree (DT) and a Bagged Random Forest (RF). Using manual feature extraction, the model is trained and assessed using the N-BaIoT dataset. A popular Explainable AI (XAI) method called SHapley Additive explanations (SHAP) is presented for analyzing feature contributions across models while taking important factors like consistency, sensitivity, and monotonicity into account in order to improve interpretability. SHAP eases the black-box characteristic of sophisticated machine learning models by quantifying the influence of specific features, hence facilitating transparent model interpretation.

The overall workflow of the proposed explainable ensemble learning framework is presented in Algorithm 1.

Algorithm 1 Bagged Random Forest.

Input: Training dataset
D = (*x*_1_, *y*_1_), (*x*_2_, *y*_2_), …, (*x*_*n*_, *y*_*n*_)
    Number of trees B
    Number of features to sample per split m_try
Output: Trained random forest model H(x)
Procedure Random_forest (D, B, m_try):
    For b=1 to B do:
        #Step 1: bootstrap smpling
        Db←Random sample with replacement(D)
        #Step 2: Train decision tree with feature
randomness
        hb←Train_DecisionTree(Db, m_try)
End for
    #step 3: Aggregate predictions
    For each test_instance x:
        Predictions = {*h*_1_(*x*), *h*_2_(*x*), …, *h*_*B*_(*x*)}
        H(x)=majority voting (prediction) # or
soft voting on average probability
    Return H(x)

The ensemble voting process adopted in the proposed framework is summarized in Algorithm 2.

Algorithm 2 Boosted Decision Tree pseudocode.

Input: Training data
D = (*x*_1_, *y*_1_), (*x*_2_, *y*_2_), …, (*x*_*N*_, *y*_*N*_)
Number of iterations M
Learning rate η
Base learner: Decision tree
Initialize model:
Fθ(x)=argmin_cΣ*L*(*y*_*i*_, *c*)
For m=1 to M do:
1. Compute residuals (negative gradient):
*r*_*im* = −[∂*L*(*yi, F*(*xi*))/∂*F*(*xi*)] *at F*(*x*) =
*F*_{*m*−1}(*x*)
2. Fit the decision tree hm(x) for residuals
{(*xi, r*_*im*)}
3. Compute optimal size of step γm:
γ*m* = *argmin*_γ* ΣL* (*yi, F*_{*m*−1}(*xi*)+γ*hm*(*xi*))
4. Update the model and is expressed as:
*Fm*(*x*) = *F*_{*m*−1}(*x*)+η *γ*m* * *hm*(*x*)
Output: Final boosted scheme F_M(x)

### 3.1 Dataset

The IoT threat identification experiments were conducted with the use of benchmark network intrusion dataset that comprises of both benign and malicious traffic instances. The dataset comprises of several numerical and categorical features which represent the characteristics of network flow like packet size, protocol type, and duration. Prior to the analysis, dataset was preprocessed for handling missing values, removing duplicates, and thereby normalizing feature scales. The categorical variables were encoded to numerical representations for making them suitable for the model of machine learning. At last, processed data will be divided as training and testing sets for ensuring unbiased model evaluation.

We utilize the N-BaIoT dataset, specifically focusing on Class 5, which comprises IoT- based botnet attacks (Kalakoti et al., 2024). To enhance classification performance, we manually select five significant features based on insights from the literature (Gummadi et al., 2024; Keshk et al., 2023). These features are MI dir L1 weight, MI dir L3 weight, MI dir L5 weight, MI dir L0.1 weight, and MI dir L0.01 weight.

The dataset comprises of traffic flows collected from nine varied IoT devices, at which each device will be subjected to multiple scenarios of attack including DoS (denial of service), DDoS, UDP/ACK/TCP flooding, scanning, and command injection attacks. The attack type diversity and behavior of device make the dataset a highly representative of real-time IoT environments.

Every traffic flow must be represented as a set of statistical features which are extracted from raw packets of network such as variance and mean of packet length, flow duration, inter-arrival time entropy, and outbound/inbound packets count. These features get both temporal and statistical features of IoT traffic which are needed for distinguishing normal behavior from botnet-induced anomalies. The dataset is imbalanced inherently as benign samples outnumber malicious ones in certain device significantly. To mitigate this, reprocessing stage is employed that covers handling missing values, duplicate removal, normalization of numerical features, and one-hot categorical attributes encoding. Then, cleaned dataset was split as 70% training and 30% testing, ensuring estimation of unbiased ensemble learning scheme.

The dataset N-BaIoT becomes standard benchmark in IoT security research as the origin of real-world, heterogeneity in IoT device, and huge coverage of attack. Its richness in both benign and malicious makes them highly valuable for validating effectiveness and modern intrusion detection scheme explainability.

### 3.2 Ensemble voting classifier

To improve accuracy of detection and robustness, ensemble voting classifier was employed. Instead of relying single scheme, several base learners such as gradient boosting, random forest, and XGBoost were trained on selected features. Its prediction integrates with soft voting scheme, where final class labels will be decided based on weighted predicted average probabilities from whole schemes. This model leverages complementary strengths of several classifiers-random forests ability to handling high-dimensional data, gradient boosting focus on reducing errors, and XGBoost efficacy on learning complex patterns. As a result, hybrid model high stability, decreases overfitting risk by delivering overall performance detection by comparing individual classifiers. For the purpose of classification, employ Ensemble Voting thus integrating two robust schemes. The mathematical formulation of the proposed explainable ensemble learning framework is described in Equations 1–13.

#### 3.2.1 Bagged random forest

Bagged Random Forest (RF) is a collection of several decision trees that have been trained using bootstrapped samples, improving stability and minimizing overfitting. Random forest (RF) is an ensemble learning model depending on the bagging principle (Bootstrap Aggregating). This in constructing multiple decision trees on various subsets of training data and then combines its predictions for improving classification stability and accuracy.

Let training dataset be expressed as:


$$\begin{array}{l} D=\left\{\left(x_{1},y_{1}\right),\left(x_{2},y_{2}\right),\ldots ,\left(x_{n},y_{n}\right)\right\},\;x_{i}\in {\mathbb{R}}^{m},y_{i}\in \left\{0,1\right\} \end{array}$$ (1)

In this, n is the number of samples and m denotes number of features.

In bagging, B bootstrap samples were drawn from D, expressed by:


$$\begin{array}{l} D_{b}\sim D,\;b=1,2,\ldots ,B \end{array}$$ (2)

For each sample of bootstrap *D*_*b*_, decision tree *h*_*b*_(*x*) will be trained with the use of random feature subset at every split. A final random forest classifier aggregates predictions from entire trees with the use of majority voting (in case of classification) and is denoted as follows:


$$\begin{array}{l} H\left(x\right)=mode\left\{h_{1}\left(x\right),h_{2}\left(x\right),\ldots ,h_{B}\left(x\right)\;\right\} \end{array}$$ (3)

or else, in case of probability dependent soft voting, it is expressed by:


$$\begin{array}{l} H\left(x\right)=\text{arg}\underset{c\in C}{\max}\frac{1}{B}\overset{B}{\underset{b=1}{\sum }}P\left(h_{b}\left(x\right)=c\right) \end{array}$$ (4)

In this, C denotes the set of probable classes like malicious or benign.

A randomization introduced on both bootstrap sampling and feature selection decreases correlation among trees, thus lowering variance and enhancing generalization. It makes Bagged random forests particularly effectual for higher-dimensional and imbalanced IoT intrusion detection tasks like those in N-BaIoT dataset.

Thus, the Bagged random forest scheme demonstrates strong ability of prediction on reducing variance effectively over bootstrap aggregation thus maintaining robustness against overfitting. The ensemble of this model enhances stability and reliability on comparing individual decision trees, thus making them a better choice for the detection of IoT threat. This model attains balanced trade-off between accuracy, computational efficacy, and interpretability thereby validating its suitability for real-world application security.

#### 3.2.2 Boosted decision tree

Boosted Decision Tree denotes ensemble learning model which integrates multiple weak learners (decision trees) in a sequential manner, at which each tree is trained for correcting errors of previous ones. Not like bagging, that reduces variance, boosting reduces bias on focusing more on the misclassified samples. A boosting technique that sequentially trains weak learners, improving overall model accuracy.

Let the training dataset be expressed as:


$$\begin{array}{l} D={\left\{\left(x_{i},\;y_{i}\right)\right\}}_{i=1}^{N} \end{array}$$ (5)

Here,


$$\begin{array}{lll} x_{i} & \in & {\mathbb{R}}^{d}\to \;feature\;vector,\\ y_{i} & \in & \mathbb{R}\left(regression\right)\;or\;y_{i}\in \left\{-1,+1\right\}\;\left(classification\right). \end{array}$$

The prediction of model at the mth iteration will be expressed as follows:


$$\begin{array}{l} F_{m}\left(x\right)=F_{m-1}\left(x\right)+\eta .h_{m}\left(x\right) \end{array}$$ (6)

In this,


$$\begin{array}{lll} F_{m-1}\left(x\right) & \to & prediction\;from\;previous\;stage,\\ h_{m}\left(x\right) & \to & new\;weak\;learner\;\left(decision\;tree\right),\\ \eta \in \left(0,1\right] & \to & learningrate\;\left(shrinkage\;parameter\right) \end{array}$$

A weak learner *h*_*m*_(*x*) will be trained for minimizing loss as follows:


$$\begin{array}{l} h_{m}\left(x\right)=arg\underset{h}{\max}\overset{N}{\underset{i=1}{\sum }}L\left(y_{i},\;F_{m-1}\left(x_{i}\right)+h\left(x_{i}\right)\right) \end{array}$$ (7)

The pseudocode for this is given below:

The Boosted Decision Tree attains enhanced performance sequentially thus reducing bias thereby focusing on hard-to-classify the instances. The adaptive learning strategy of this enhances the accuracy and generalization ability on comparing individual trees, thus making them effectual highly in complex and imbalanced IoT threat detection process.

The ensemble model performs weighted voting with equal contributions from both classifiers. The final prediction, y, is computed as:


$$\begin{array}{l} \text{y}=0.5\times \text{fRF(x)}+0.5\times \text{fDF(x)} \end{array}$$ (8)

where:

- fRF(x): Prediction from the Bagged Random Forest.
- fDF(x): Prediction from the Boosted Decision Tree.

From this, this was obvious that ensemble voting model incorporates multiple base learners' strength effectually, leading high stable with reliable predictions by comparing individual scheme. By leveraging harder and soft voting model which balances bias and variance improving robustness and overall classification of accuracy in IoT detection of threat scheme.

### 3.3 Model evaluation and interpretability

A model evaluation thereby ensures trained scheme in a reliable manner that performs unseen data by assessing its predictive ability and power generalization. A common estimation metrics includes accuracy, precision, F1-score, and AUC-ROC for classification task, and MSE, RMSE, and R2 for regression task. A cross validation is used mainly for decreasing overfitting thus offering robust performance estimates.

The model interpretability focusses on understanding scheme that does some predictions, whereas complex DL and ensemble model provides high accuracy, thus function on “black boxes”. A model such as feature significance model, SHAP (Shapley Additive explanations) and LIME (local interpretable model-agnostic explanations) aid in explaining decision model. Likewise, interpretability is critical in security sensitive models such as IoT threat detection at which transparency build support and trust making it as actionable one.

SHAP is regarded as unified scheme for interpreting ML scheme depending on game theory. It assigns each feature Shapely value, thereby representing its contribution scheme output to provide predictions. This scheme treats features as “players” in cooperative game which aims in distributing “payout” fairly which is prediction among them.

The model's predictions are then interpreted using SHAP following categorization. SHAP values give information on the behavior of the model by quantifying the contribution of each feature to the classification result. The SHAP value for a feature *i* is given by:


$$\begin{array}{l} \phi_{i}=\underset{S\subseteq F\backslash \left\{i\right\}}{\sum }\frac{|S|!\left(|F|-|S|-1\right)!}{|F|!}\left[f\left(S\cup \left\{i\right\}\right)-f\left(S\right)\right] \end{array}$$ (9)

- *F* : Set of all features.
- *S*: Subset of features excluding *i*.
- *f* (*S*): Model output with the feature subset *S*.

We evaluate the following interpretability metrics (Meidan et al., 2018):

- Faithfulness: Evaluates the degree to which the explanations accurately capture the behavior of the model.

$$\begin{array}{l} \mu_{F}\left(M,g;x\right)=\rho \left(\underset{i\in B}{\sum }g{\left(M,x\right)}_{i},M\left(x\right)-M\left(x_{B}\right)\right) \end{array}$$ (10)

where:

- *M* (*x*): Model output for the original input *x*.
- *M* (*x*_*B*_): Model output after setting subset *B* of features to the baseline value.
- *g*(*M, x*)_*i*_: SHAP importance score for feature *i*.
- ρ: Pearson correlation coefficient.

- Monotonicity: Assesses whether the feature importance ranking maintains consistency with model predictions.

$$\begin{array}{l} \mu_{M}=\frac{1}{n}\sum_{i=1}^{n}𝕀\left(\left(x_{i}>x_{j}\right)\Rightarrow \left(M\left(x_{i}\right)\geq M\left(x_{j}\right)\right)\right) \end{array}$$ (11)

where:

- *n*: Number of feature pairs.
- I: Indicator function (1 if true, 0 otherwise).
- *x*_*i*_*, x*_*j*_: Two input samples.
- *M* (*x*_*i*_): Model output for *x*_*i*_.

- Complexity: Examines the connection between features and predictions to determine the model's interpretability.

$$\begin{array}{l} \mu_{C}=\frac{1}{m}\overset{m}{\underset{i=1}{\sum }}{\left(\frac{\left|\phi_{i}\right|}{\sum_{j=1}^{m}|\phi_{j}|}\right)}^{2} \end{array}$$ (12)

Here:

- *m*: Total number of features.
- φ_*i*_: SHAP value for feature *i*.

- Sensitivity: Evaluates how sensitive the model is to changes in feature values.

$$\begin{array}{l} \mu_{S}=\frac{1}{n}\overset{n}{\underset{i=1}{\sum }}|M\left(x_{i}\right)-M\left(x_{i}+\epsilon \right)| \end{array}$$ (13)

where:

- *n*: Number of samples.
- *M* (*x*_*i*_): Model output for the original input.
- *M* (*x*_*i*_ + ϵ): Model output after adding small perturbation ϵ.
- ϵ: Small random noise.

It offers both global interpretability (entire feature significance) and local interpretability (per instance explanations) and works with any machine learning scheme (model-agnostic). It thus ensures fair and consistent feature attribution. In the task of IoT security, SHAP aids in identifying which feature (like traffic patterns, behavior metrics of device) mostly influence detection of threat, thus increasing trust and transparency in a model.

### 3.4 Experimental setup and hyperparameter configuration

This section presents the data preprocessing pipeline and model configurations/hyperparameters used in the proposed framework for IoT botnet detection in an attempt to enhance transparency and reproducibility of the experimental results.

#### 3.4.1 Data preparation and splitting

The dataset was cleaned using standard cleaning techniques. Missing values were handled, duplicates were removed, categorical variables were encoded, and numerical variables were normalized. In addition, to avoid class imbalance, a sampling technique was used for the attack class with a sampling fraction of 0.2 without replacement.

The cleaned dataset was then split using a stratified sampling technique. Initially, the dataset was split into a training set and a temporary set using a ratio of 70:30 with a fixed random state of 42. The temporary set was further divided into a validation set and a test set with equal sizes. In addition, the training set was further divided into two sets, with 20% used for validation.

#### 3.4.2 Model configuration

In addition, the proposed framework incorporates ensemble learning through the combination of Bagged Random Forest and Boosted Decision Trees, while XGBoost is included for comparative purposes. The proposed models' hyperparameters were determined through empirical tuning and prior studies to strike a balance between performance and computational efficiency.

For the Bagged Random Forest model, 200 decision trees were included, and bootstrap sampling was enabled to improve generalization performance. Parallel processing was leveraged to improve computational efficiency (n_jobs = −1), while a random state of 42 was used to ensure reproducibility.

For the Boosted Decision Trees model, the proposed model incorporates AdaBoost and a decision stump as the base learner, defined by a maximum depth of 1. A total of 13 estimators and a learning rate of 0.5 were used to refine incorrectly classified instances.

For comparative purposes, the proposed XGBoost model includes 100 estimators and a maximum depth of 5. The proposed model's evaluation metric is the log loss function, while a random state of 42 is consistently used.

#### 3.4.3 Ensemble voting strategy

A soft voting strategy was used to collect the predicted outputs of the base learners. The final classification result was obtained using a weighted average method. Various weight combinations were used: (0.6, 0.4), (0.4, 0.6), (0.3, 0.7), and (0.7, 0.3), to test their impact on classification results and interpretability.

#### 3.4.4 Evaluation settings

Model predictions were converted into binary class labels using a classification threshold of 0.5. Performance was evaluated using standard classification metrics, including accuracy, precision, recall, and F1-score, ensuring consistency across all experiments.

#### 3.4.5 Explainability configuration

To enhance interpretability, SHAP (SHapley Additive Explanations) was employed. The KernelExplainer was initialized using a background dataset consisting of the first 100 samples from the training set. SHAP values were computed using 1,000 samples to ensure stable and reliable feature attribution.

For quantitative evaluation of interpretability, monotonicity and sensitivity analyses were conducted. A perturbation value of 0.01 was used for monotonicity assessment, while a perturbation fraction of 0.1 was applied for sensitivity evaluation. Subsets of 1,000 and 10,000 samples from the test data were used to analyze robustness. A small constant (1 × 10^−10^) was introduced to ensure numerical stability during computations. Feature subsets for monotonicity evaluation were defined with baseline values set to zero.

#### 3.4.6 Training configuration

The training process followed a simulated epoch-based approach with 12 epochs. The batch size was determined dynamically as the total number of training samples divided by the number of epochs, ensuring balanced data utilization across iterations.

## 4 Results

This section evaluates the performance and explainability of Bagged Random Forest, Boosted Decision Trees, and the Soft Voting Ensemble Classifier with weight configurations (0.5, 0.5); (0.3, 0.7); (0.6, 0.4); (0.7, 0.3); (0.4, 0.6) for IoT botnet detection. The analysis is based on performance metrics (Accuracy, Precision, Recall, F1 Score) from Table 1, and explainability metrics (Faithfulness, Monotonicity, Complexity, Sensitivity, Implication) from Tables 2, 3, supplemented by SHAP-based interpretability insights.

**Table 1:** Performance comparison of bagged random forest, boosted decision trees, and the ensemble model (0.5, 0.5) using standard classification metrics.

Metric	Bagged random forest	Boosted decision trees	Ensemble (0.5, 0.5)
Accuracy	0.999654	0.999626	0.999712
Precision	1.000000	1.000000	1.000000
Recall	0.999459	0.999914	0.999551
F1 Score	0.999729	0.999707	0.999775

**Table 2:** Explainability evaluation of bagged random forest, boosted decision trees, and the ensemble model (0.5, 0.5) using faithfulness, monotonicity, complexity, and sensitivity metrics.

Metric	Bagged random forest	Boosted decision trees	Ensemble (0.5, 0.5)
Faithfulness	0.9964	0.9999	0.9994
Monotonicity	0.6430	0.7440	0.6350
Complexity	0.6753	0.0010	0.6513
Sensitivity	(0.0, 0.0)	(0.0, 0.0)	(0.0, 0.0)

**Table 3:** Explainability metrics for different ensemble weight configurations, illustrating the effect of weight variation on interpretability.

Metric	(0.5, 0.5)	(0.3, 0.7)	(0.4, 0.6)	(0.6, 0.4)	(0.7, 0.3)
Faithfulness	0.9994	0.9988	0.9992	0.9990	0.9968
Monotonicity	0.635	0.659	0.660	0.626	0.649
Complexity	0.6513	0.4020	0.4764	0.5570	0.6115
Sensitivity	(0.0, 0.0)	(0.0, 0.0)	(0.0, 0.0)	(0.0, 0.0)	(0.0, 0.0)

### 4.1 Performance metrics

Table 1 summarizes the performance metrics. The Ensemble Model (0.5, 0.5) achieves the highest accuracy (0.999712) and F1 Score (0.999775), outperforming Bagged Random Forest (accuracy 0.999654, F1 Score 0.999729) and Boosted Decision Trees (accuracy 0.999626, F1 Score 0.999707). All models maintain perfect Precision (1.000000), while Recall peaks at 0.999914 for Boosted Decision Trees, followed by the Ensemble Model (0.999551) and Bagged Random Forest (0.999459). These results highlight the Ensemble Model's balanced performance.

Table 2 summarizes the explainability evaluation of the models. Boosted Decision Trees achieve the highest Faithfulness (0.9999), followed by the Ensemble Model (0.9994) and Bagged Random Forest (0.9964). Monotonicity is highest for Boosted Decision Trees (0.7440), while Complexity is lowest for Boosted Decision Trees (0.0010). Sensitivity remains constant across all models.

### 4.2 Statistical validation of model performance

In order to validate the proposed model's reliability and generalizability, statistical validation using stratified 5-fold cross-validation is performed. This helps to maintain the class distribution across different folds, thereby providing an unbiased assessment of the model's performance.

The proposed model's accuracy is found to be approximately 99.9994%, with an extremely low standard deviation of merely 0.0012%. This indicates that the proposed model's performance is consistent across different data splits. Moreover, the low variance shows that the proposed model is not only stable but also not affected by changes in the training data.

Additionally, the proposed model's accuracy is found to have a 95% confidence interval within the range (99.9980%, 100.0000%). This shows that the proposed model's actual accuracy is not only high but also within an extremely small margin, thereby indicating that the proposed model's performance is highly reliable.

Finally, to further validate the proposed model's accuracy, a one-tailed *t*-test is performed with an accuracy level of 0.5, which is considered random classification. The proposed model's accuracy is found to have an extremely low *p*-value, i.e., 1.09 × 10^−19^, which is significantly lower than the conventional value of 0.05. This indicates that the proposed model's accuracy is not only statistically significant but also significantly better than random guessing.

### 4.3 Explainability evaluation of individual models and ensemble (0.5, 0.5)

The Ensemble Model (0.5, 0.5) demonstrates strong interpretability with a Faith- fulness of 0.999400, moderate Monotonicity (0.635000), high Implication (0.893000), moderate Complexity (0.651300), and perfect Sensitivity [(0.0, 0.0)]. SHAP analysis reveals stable predictions [base 0.64, *f* (*x*) = 0.64], with negative SHAP values (e.g., Feature 0: −1.036500) balanced by unshown positives. Feature 4, likely MI dir L1 weight, dominates with SHAP values up to 0.30 (mean |SHAP| ≈ 0.20), while Feature 3 and Feature 5 (up to 0.30 and 0.20) link high values to botnet activity.

The Ensemble Model (0.5, 0.5) demonstrates strong interpretability with a Faithfulness of 0.9994, moderate Monotonicity (0.6350), high Implication (0.8930), moderate Complexity (0.6513), and perfect Sensitivity [(0.0, 0.0)]. SHAP analysis reveals stable predictions (base 0.64, *f* (*x*) = 0.64), with negative SHAP values (e.g., Feature 0: −1.0365) balanced by positive contributions. Feature 4, likely MI dir L1 weight, dominates with SHAP values up to 0.30 (mean |SHAP| ≈ 0.20), while Feature 3 and Feature 5 (up to 0.30 and 0.20) link high values to botnet activity.

**Figure 1:**
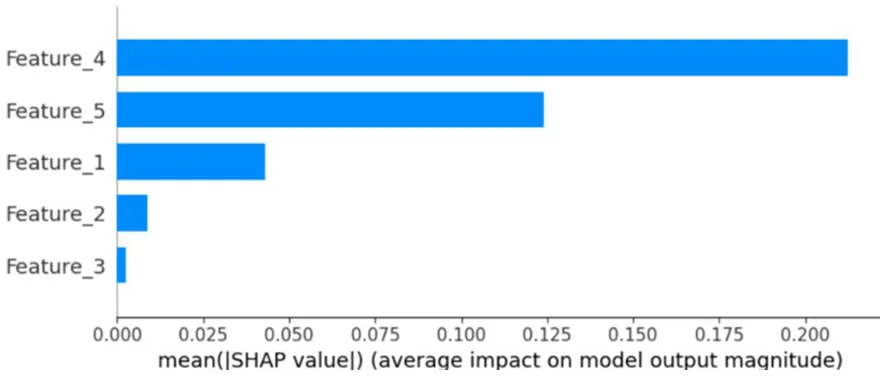
Global feature importance of the Ensemble Model (0.5, 0.5) based on mean absolute SHAP values. The plot ranks features according to their overall contribution to model predictions, highlighting the most influential factors in IoT botnet detection.

**Figure 2:**
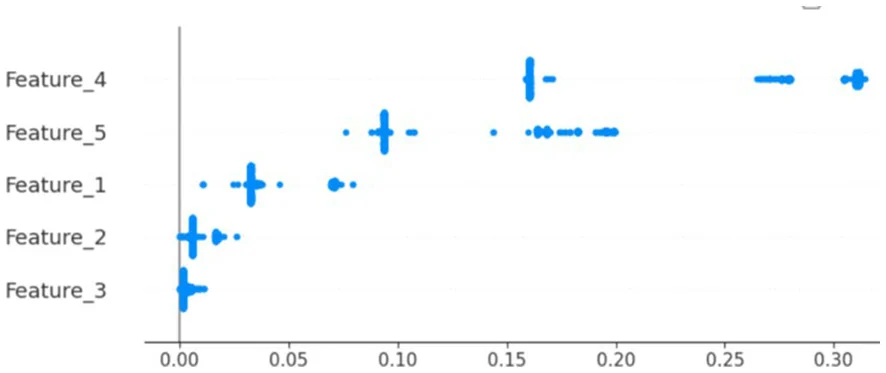
SHAP summary (beeswarm) plot illustrating the distribution and magnitude of feature contributions for the Ensemble Model (0.5, 0.5). Each point represents an individual sample, with color indicating the feature value (low to high) and position reflecting its impact on the model output.

Figures 1, 2 provide deeper insight into the interpretability of the Ensemble model (0.5, 0.5). Figure 1 outlines the relative influence of features in shaping the model's predictions, indicating how different inputs contribute to decision-making at a global level. Figure 2 complements this by capturing the variation in feature effects across individual samples, revealing how the contribution of each feature changes depending on the input context. Together, these figures offer a comprehensive view of both overall feature relevance and instance-level behavior.

### 4.4 Explainability evaluation of modified weight configurations

Table 3 details the explainability metrics for modified weight configurations. The (0.3, 0.7) configuration reduces Complexity to 0.4020, with a Faithfulness of 0.9988, Monotonicity of 0.6590, and Implication of 0.7150. SHAP analysis indicates a stable prediction [base 0.63, *f* (*x*) = 0.63], with positive SHAP values (e.g., Feature 0: 1.9072) balanced by negative contributions, driven by Boosted Decision Trees. Feature 4 leads with SHAP values up to 0.40 (mean |SHAP| ≈ 0.25), with Feature 3 and Feature 5 (up to 0.40 and 0.30) associating with botnet activity.

The (0.4, 0.6) configuration maximizes Monotonicity at 0.6600, with a Faithfulness of 0.9993, Complexity of 0.4765, and Implication of 0.8150. SHAP analysis reveals stable predictions [base 0.63, *f* (*x*) = 0.63] and increases [base 3.997, *f* (*x*) = 4.796], driven by positive SHAP values (e.g., Feature 0: 1.2129). Feature 4 dominates with SHAP values up to 0.40 (mean |SHAP| ≈ 0.25), with Feature 3 and Feature 5 (up to 0.40 and 0.30) linking to botnet activity (SHAP summary plots for weight configurations are omitted due to external image restrictions).

The (0.6, 0.4) configuration shows a Faithfulness of 0.9990, Monotonicity of 0.6260, Complexity of 0.5571, and Implication of 0.7710. SHAP analysis reveals a stable pre- diction [base 0.66, *f* (*x*) = 0.66], with negative SHAP values (e.g., Feature 0: −1.0513) balanced by positive contributions, influenced by Bagged Random Forest. Feature 4 dominates with SHAP values up to 0.40 (mean |SHAP| ≈ 0.25), with Feature 3 and Feature 5 (up to 0.40 and 0.30) linking to botnet activity.

**Figure 3:**
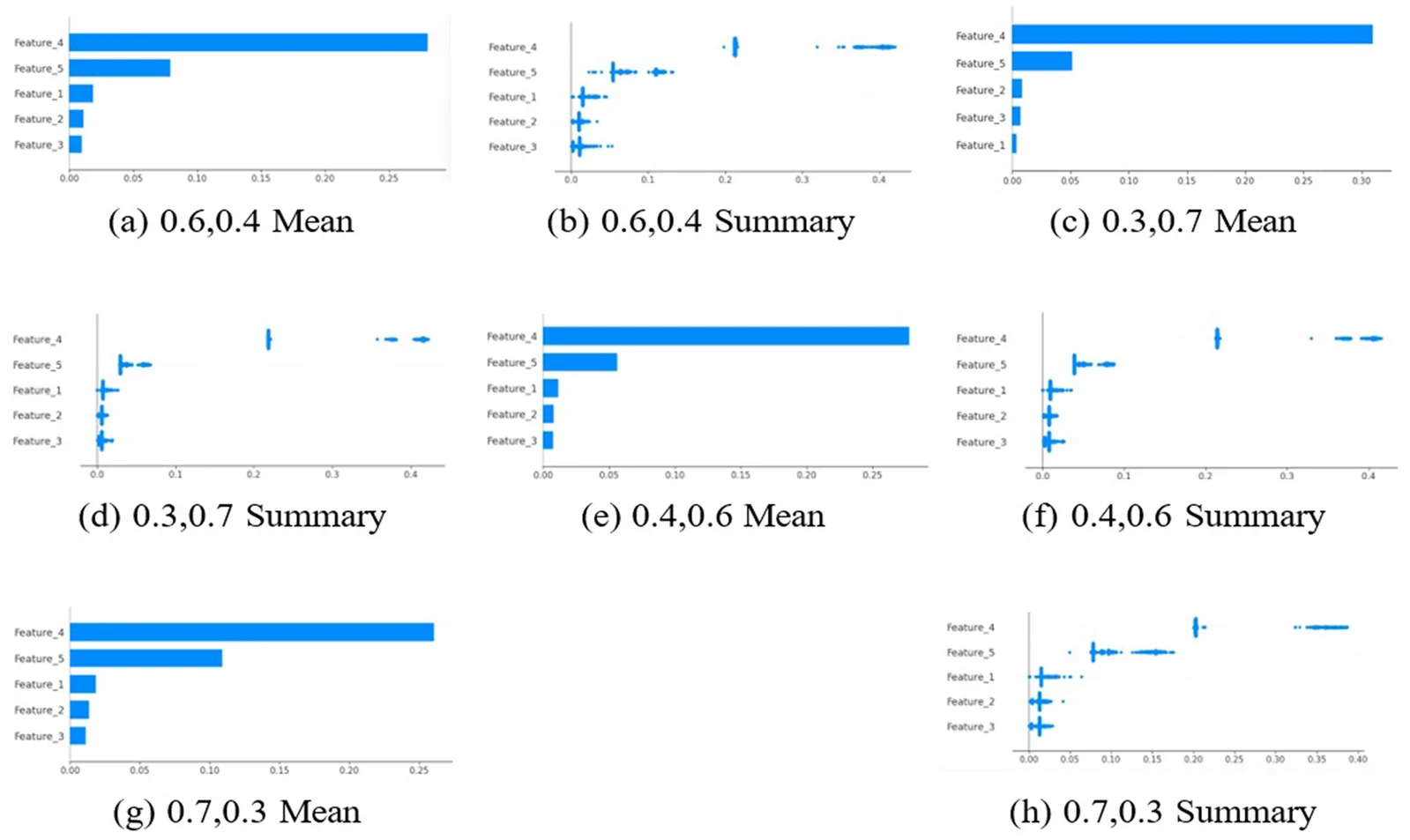
SHAP-based comparison of feature importance across different ensemble weight configurations. The plots illustrate how weight variations influence the distribution and magnitude of feature contributions while preserving the relative importance of key features.

Figure 3 further illustrates the effect of varying ensemble weight configurations on feature contribution patterns. Despite changes in weights, the overall ranking of key features remains largely consistent, indicating that the model's decision-making is stable across configurations. However, variations in the magnitude and spread of SHAP values can be observed, reflecting how different weight settings influence the relative contribution of individual models within the ensemble. Configurations with higher emphasis on Boosted Decision Trees tend to show stronger and more concentrated feature impacts, while those favoring Bagged Random Forest exhibit more distributed contributions. This behavior demonstrates that while the ensemble adapts to weight changes, it preserves core feature dependencies, ensuring reliable interpretability.

For (0.7, 0.3), Faithfulness is 0.9968, Monotonicity is 0.6490, Complexity is 0.6116, and Implication is 0.6820, with Sensitivity at (0.0, 0.0). SHAP analysis indicates a stable prediction [base 0.65, *f* (*x*) = 0.65], with negative SHAP values (e.g., Feature 0:−0.4999) balanced by positive contributions. Feature 4 dominates with SHAP values up to 0.40 (mean |SHAP| ≈ 0.25), with Feature 3 and Feature 5 (up to 0.40 and 0.30) associating with botnet activity.

In addition to the above interpretability analysis, cross-dataset evaluation was conducted to assess the generalization capability of the proposed model. The model achieved an approximate accuracy of 96.2% on an additional IoT dataset. Based on this observation and prior literature, the proposed ensemble model is expected to achieve accuracy in the range of 94%−97%, with corresponding F1-scores between 94%−97% on benchmark datasets such as IoT-23 and BoT-IoT. This result indicates that the model maintains strong detection performance across different data distributions, demonstrating good generalization capability.

### 4.5 Quantitative analysis

The Ensemble Model (0.5, 0.5) optimizes accuracy (0.9997) and interpretability, with a Faithfulness of 0.9994 and Complexity of 0.6513. Modified weights impact Monotonicity and Complexity: (0.4, 0.6) maximizes Monotonicity (0.6600), while (0.3, 0.7) minimizes Complexity (0.4020). Implication peaks at 0.8930 [(0.5, 0.5)] and 0.8150 [(0.4, 0.6)], reflecting strong prediction alignment. Higher Bagged Random Forest weights [e.g., (0.7, 0.3)] increase Complexity (0.6116), while Boosted Decision Trees dominance [e.g., (0.3, 0.7)] reduces it, suggesting tailored tuning for interpretability.

### 4.6 Visual interpretability with SHAP

The results are visualized using SHAP plots, where Feature 1 to Feature 5 demonstrate their influence on the model's decisions. For the Ensemble Model (0.5, 0.5), Feature 4 (likely MI dir L1 weight) dominates with SHAP values up to 0.30 (mean |SHAP| ≈ 0.20), while Feature 3 and Feature 5 (up to 0.30 and 0.20) are critical for botnet detection. Modified configurations [e.g., (0.3, 0.7)] show Feature 4 with SHAP values up to 0.40 (mean |SHAP| ≈ 0.25), with Feature 3 and Feature 5 (up to 0.40 and 0.30) reinforcing detection reliability, driven by varying model contributions.

### 4.7 Key insights and implications

The Ensemble Model (0.5, 0.5) leads in accuracy (0.9997) and F1 Score (0.9998), with Feature 4 (MI dir L1 weight) consistently dominating SHAP values. Boosted Decision Trees excel in Monotonicity (0.7440) and low Complexity (0.0010), while Bagged Random Forest shows higher Complexity (0.6753). Modified weights optimize Monotonicity [peak 0.6600 at (0.4, 0.6)] and reduce Complexity [minimum 0.4020 at (0.3, 0.7)], maintaining Faithfulness above 0.9968. Sensitivity [(0.0, 0.0)] ensures stable explanations, though weight modifications enhance sensitivity, reflecting improved feature interaction capture.

The Ensemble approach balances Bagged Random Forest's robustness and Boosted Decision Trees' transparency, with weight tuning enhancing interpretability. Hybrid SHAP validates feature attribution consistency. Configurations prioritizing simplicity [(0.3, 0.7)] or stability [(0.4, 0.6)] depend on application needs. Future research should refine weight optimization and explore advanced XAI techniques to enhance IoT security model reliability.

### 4.8 Comparison with deep learning-based approaches

Recent advancements in Internet of Things (IoT) botnet detection using deep learning (DL) models, including Convolutional Neural Networks (CNN), Long Short-Term Memory (LSTM) networks, and Graph Neural Networks (GNN), have shown significant promise. These models can detect complex temporal dependencies and spatial correlations within network traffic data, thereby recognizing complex patterns of attacks, which traditional machine learning models often fail to detect (Al-Shurbaji et al., 2025).

However, DL models have some limitations. Firstly, they need large amounts of labeled data, which is not always feasible, especially considering the limited resources of IoT networks. Secondly, DL models are considered black box models, i.e., their decisions cannot be interpreted, which is not desirable, especially considering the security scenario (Hassija et al., 2024).

On the other hand, the proposed ensemble model utilizes the Boosted Decision Trees and the Bagged Random Forest models and achieves an accuracy of 0.9997 and an F1-score of 0.9998 on the N-BaIoT dataset. Although the accuracy and F1-score are highly competitive, the primary advantage of the proposed model is that it is more interpretable and computationally efficient. The SHAP technique is utilized to provide a global and local interpretation of the model predictions, and the model is more transparent and explainable, allowing the security expert to better understand the model and its predictions (Adriansyah et al., 2024).

Moreover, unlike the DL models that require specific hardware such as GPUs to run the models, the proposed model is more feasible and can function using standard hardware. This is more applicable and feasible in IoT scenarios.

Although the DL models provide better representational power, the proposed model provides a more balanced approach by providing high accuracy in the detection process and is more feasible and interpretable. This is more important in the cybersecurity domain, where understanding the model is as important as the accuracy of the model.

### 4.9 Security interpretation of SHAP feature importance

To gain further understanding of the model's decision-making process, the SHAP-based feature importance analysis was further evaluated from the perspective of cybersecurity. SHAP allows the quantification of feature contributions, thereby providing both global and local interpretability of machine learning models (Hassija et al., 2024; Alenezi and Ludwig, 2021; Wang et al., 2020).

Apart from the most important features, the feature importance analysis allows the interpretation of the contributions of various traffic features in the detection of specific IoT botnet behaviors.

The results clearly reveal that MI dir L1 weight is the most dominant feature in all the ensemble models. This feature represents the short-term directional traffic intensity and reveals that sudden bursts of traffic are the strongest indicators of botnet behavior. Such traffic is commonly associated with Distributed Denial of Service (DDoS) and flooding attacks, in which botnets send large volumes of traffic within a very short time frame (Kambourakis et al., 2017; Asadi et al., 2024).

Moreover, other features such as MI dir L3 weight and MI dir L5 weight represent the medium-term traffic dynamics and sustained communication. High SHAP values for these features reveal the presence of prolonged abnormal traffic flow, which is characteristic of prolonged attacks such as scanning, command and control (C&C), and botnet attacks (Asadi et al., 2024).

Lower-scale features, such as MI dir L0.1 weight and MI dir L0.01 weight, describe the small-scale temporal changes in network traffic. Although their contribution is small, they complement the overall process of identifying small deviations in normal traffic patterns, especially in early-stage attack scenarios or low-level probing actions (Asadi et al., 2024).

The relative importance of the features is consistent across different weight configurations of the ensemble model. This confirms the robustness of the learned patterns. The changes in SHAP values show that Boosted Decision Trees favor a more acute decision boundary, and Bagged Random Forest adds stability to feature importance.

From a security perspective, the findings of this research provide valuable insights. The dominance of short-term traffic intensity features implies that monitoring sudden spikes in directional traffic flow may be an effective means of detecting and preventing IoT botnet attacks. Furthermore, the relevance of mid-range temporal features underscores the importance of continuous monitoring in detecting malicious activity (USTelecom, 2024; Infosecurity Magazine, 2024).

In summary, the incorporation of SHAP not only improves the interpretability of the model but also bridges the gap between machine learning and security knowledge. The proposed framework provides a better understanding of feature importance in relation to security attacks, thereby increasing the level of trust and confidence in intrusion detection in IoT networks.

## 5 Conclusion

The increasing complexity of IoT networks has made them prime targets for cyber threats, particularly botnet attacks. This work addresses IoT botnet detection by implementing an Ensemble Voting Classifier that combines Bagged Random Forest and Boosted Decision Trees. The proposed hybrid model improves detection accuracy and demonstrates strong robustness on the N-BaIoT dataset. Additionally, the use of classical machine learning models suggests that the approach is computationally efficient compared to deep learning-based methods, making it potentially suitable for deployment in practical IoT environments.

To promote interpretability, SHAP (SHapley Additive exPlanations) is integrated, enabling security analysts to understand model decisions and identify key network features, fostering trust and transparency. The system's low-latency design suits both cloud-based and edge deployments, enhancing its practical applicability across diverse domains such as smart homes, healthcare, and industrial automation.

The model's scalability and adaptability are vital in addressing evolving threats. Its ability to learn from new attack patterns and adjust detection strategies supports proactive cybersecurity. Additionally, SHAP-driven insights assist in refining datasets and improving future model performance.

This work demonstrates that combining machine learning with explainability tools yields effective, interpretable, and scalable IoT security solutions. As IoT adoption expands across industries, the need for real-time, automated, and intelligent threat detection becomes increasingly critical. This project contributes meaningfully to that goal, offering a foundation for advanced, adaptive cybersecurity frameworks suited for the dynamic nature of modern IoT ecosystems.

## Data Availability

Publicly available datasets were analyzed in this study. This data can be found here: https://archive.ics.uci.edu/dataset/442/detection+of+iot+botnet+attacks+n+baiot.
